# Lack of Knowledge about Sexually Transmitted Diseases (STDs): Implications for STDs Prevention and Care among Dermatology Patients in an Urban City in Vietnam

**DOI:** 10.3390/ijerph16061080

**Published:** 2019-03-26

**Authors:** Sau Huu Nguyen, Anh Kim Dang, Giang Thu Vu, Cuong Tat Nguyen, Thu Hoai Thi Le, Nu Thi Truong, Chi Linh Hoang, Tung Thanh Tran, Tung Hoang Tran, Hai Quang Pham, Nam Gia Dao, Bach Xuan Tran, Carl A. Latkin, Cyrus S. H. Ho, Roger C. M. Ho

**Affiliations:** 1 National Hospital of Dermatology and Venereology, Hanoi 100000, Vietnam; nguyenhuusau@yahoo.com (S.H.N.); lethu2203@gmail.com (T.H.T.L.); 2 Department of Dermatology and Venereology, Hanoi Medical University, Hanoi 100000, Vietnam; 3 Institute for Global Health Innovations, Duy Tan University, Da Nang 550000, Vietnam; kimanh.ighi@gmail.com (A.K.D.); cuong.ighi@gmail.com (C.T.N.); qhai.ighi@gmail.com (H.Q.P.); 4 Center of Excellence in Evidence-Based Medicine, Nguyen Tat Thanh University, Ho Chi Minh City 700000, Vietnam; giang.coentt@gmail.com (G.T.V.); tung.coentt@gmail.com (T.T.T.); gianam.coentt@gmail.com (N.G.D.); 5 Center of Excellence in Behavioral Medicine, Nguyen Tat Thanh University, Ho Chi Minh City 700000, Vietnam; nu.coentt@gmail.com (N.T.T.); chi.coentt@gmail.com (C.L.H.); 6 Institute of Orthopaedic and Trauma Surgery, Vietnam—Germany Hospital, Hanoi 100000, Vietnam; tranhoangtung.vd@gmail.com; 7 Institute for Preventive Medicine and Public Health, Hanoi Medical University, Hanoi 100000, Vietnam; 8 Johns Hopkins Bloomberg School of Public Health, Baltimore, Maryland, MD 21205, USA; carl.latkin@jhu.edu; 9 Department of Psychological Medicine, National University Hospital, Singapore 119074, Singapore; cyrushosh@gmail.com; 10 Department of Psychological Medicine, Yong Loo Lin School of Medicine, National University of Singapore, Singapore 119077, Singapore; pcmrhcm@nus.edu.sg; 11 Biomedical Global Institute of Healthcare Research & Technology (BIGHEART), National University of Singapore, Singapore 119228, Singapore

**Keywords:** STIs, STDs, sexually transmitted infections, dermatology, Vietnam

## Abstract

Sexually transmitted diseases (STDs) are a substantial global burden of diseases, especially in developing countries. Lack of awareness of STDs may lead to a delay in treatment. This study aimed to assess knowledge about STDs and the associated factors among dermatological patients. A cross-sectional study was conducted among 622 patients at Vietnam National Hospital of Dermatology and Venereology (NHD). Structured questionnaires were used to investigate the knowledge about STDs. A multivariate Tobit regression was employed to determine factors associated with knowledge about STDs. The percentage of patients knowing that syphilis was an STD was highest (57.8%), followed by herpes warts (57.7%) and HIV/AIDS (57.4%). By contrast, 26.6% and 17.2% of patients knew that chlamydia and hepatitis C were STDs. The most commonly stated symptom of STDs was purulent genital (53.5%). Nearly two-thirds of participants were aware of the curability of STDs, and 34.7% knew about vaccines for STDs. Living with partners, young age, and acquired knowledge of STDs via the Internet, social networks, and health staff were positively related to having better knowledge about STDs. Based on the results of this study, peer education, informal conversations within clusters, mass community campaigns through the Internet and social networks, and the use of online health care providers should be promoted in order to improve awareness of STDs.

## 1. Introduction

Sexually transmitted diseases (STDs) are considered one of the major causes for the global burden of diseases. According to a report from the World Health Organization (WHO), there are approximately one million new cases of sexually transmitted diseases (STDs) every day worldwide [1]. In addition, each year it is estimated that there are 357 million new infections consisting of one of four types of STDs including chlamydia, gonorrhea, syphilis, and trichomoniasis [1,2]. Beyond the immediate impact of the infections, STDs may have severe repercussions on physical health as well as the psychological and social well-being of patients [3]. Mother-to-child transmissions of STDs can occur during pregnancy and childbirth. Syphilis in pregnancy can lead to stillbirth, neonatal deaths, congenital deformities as well as increasing risk of dying from prematurity [1,4]. Women experiencing Human Papillomavirus (HPV) infection may suffer from cervical cancer as a result, and women with gonorrhea and chlamydia are at higher risk of undergoing pelvic inflammatory disease, female infertility, and preterm delivery [1,3].

However, the management of STDs is still limited due to asymptomatic or mild cases that case can be make detection difficult [5]; the diversity of pathogens, and social stigma on patients [1,6]. The imperative for enhancing knowledge of STDs is a strategic measure of the WHO to address the burden of disease [3,7]. A previous study conducted among young students indicated that most of them had heard about sexually transmitted infection (STIs), but primarily human immunodeficiency virus/acquired immunodeficiency syndrome (HIV/AIDS) rather than other types of STDs. It was reported that the students mainly obtained STD information through the Internet, newspapers, or magazines [7]. Moreover, many people do not perceive that they are at risk of becoming infected by STDs and do not have adequate knowledge about STDs, especially in developing countries [8]. Acquiring adequate knowledge of symptoms and about the prevention of STDs is critical in order to reduce the risk of sexual transmission and the prevalence of STDs [9].

The prevalence of STDs in urban areas and megacities is increasing [10]. Previous studies have shed light on the association between the spreading of STDs and mass urbanization and migration [10,11,12]. Additionally, among developing countries, STIs have been shown to directly negatively influence reproductive health and indirectly increase the risk of sexual transmission of HIV and have a heavy impact on both morbidity and mortality rates [13]. Because many symptoms of STDs are presented on the skin, a large proportion of patients showing signs of STDs tend to visit Dermatology and Venereology Hospital [14]. In Vietnam, the prevalence of STDs (excluding HIV/AIDS) has risen rapidly in the last ten years and reached 17.3% in 2017, resulting in approximately 28,654 disability-adjusted life years [15]. This study aimed to assess knowledge about STDs as well as the associated factors among dermatological patients in an urban city in Vietnam (a developing country).

## 2. Materials and Methods

### 2.1. Study Design and Setting

Our cross-sectional study was conducted at the Vietnam National Hospital of Dermatology and Venereology (NHD) from September to November 2018. NHD is the specialized national hospital that is the leader in dermatology in the country, and this is the main center for research, diagnosis, treatment, prevention, and functional rehabilitation of STDs across different illness severity levels. The techniques and tests performed at the hospital follow the standard procedures and protocols provided or recommended by the Ministry of Health, WHO, and CDC (Centers for Disease Control and Prevention).

A convenience sampling technique was used to choose participants for the study among the pool of patients attending the hospital. The eligibility criteria for selecting participants were (1) aged 18 years old or above; (2) having the ability to answer the questionnaire coherently; (3) agreeing to be involved in the study by providing written consent. Participants suffering from serious illnesses during the interview time were excluded. All participants were clearly informed about the purpose, benefits, and disadvantages of the study prior to becoming involved in the study. Upon agreeing to participate in the study, all participants signed a written informed consent form. Participants were invited into a private room at the hospital in order to ensure the confidentiality of their responses as well as the quality of the answers. Six hundred and twenty-two patients agreed to participate in the study.

### 2.2. Measurements and Instruments

A pilot study was conducted among 20 patients in August 2018. Only a few comments regarding logic and wording of the questionnaire were noted and corrected before conducting the survey. Participants were invited to complete 20-minute face-to-face interviews using structured questionnaires. The individuals who collected data were well-trained medical students at Hanoi Medical University. Data regarding the socioeconomic status of participants and their knowledge about STDs are specified below.

#### 2.2.1. Socioeconomic Characteristics

Patients self-reported data about gender, age, educational level, marital status, occupation, living area, age, monthly income, and health insurance.

#### 2.2.2. Sexually Transmitted Disease (STD)-Related Characteristics

We collected information about each participant’s current diagnosis of STDs and about whether they had ever suffered from any STDs or venereal diseases, especially STDs presented on the skin.

#### 2.2.3. Knowledge Regarding Sexually Transmitted Diseases (STDs)

Seven multiple choice questions were asked to assess participants’ knowledge of STDs. Regarding types of common STDs, signs and symptoms of STDs, and measures to prevent STDs, participants were able to choose more than one answer. We prepared a list of featured signs and symptoms of STDs and participants were queried about whether they knew each of them. Participants were asked to choose one correct answer only about the curability and availability of vaccinations for STDs. Sources of information on the prevention of STDs were also examined.

### 2.3. Statistical Analysis

Data was analyzed by STATA version 15.0 (StataCorp. LP, College Station, TX, USA). Descriptive statistics were utilized to examine the socioeconomic variables and STD-related characteristics. For each correct answer on STDs knowledge 1 point was scored. The total score of STDs knowledge was calculated by summing all correct answers. A multivariate Tobit regression model was employed to determine associated factors with knowledge about STDs (censored continuous variable). Independent variables were socioeconomic characteristics (age, gender, marital status, educational level, occupation, income level, and living areas), and methods by which the participants accessed information on the prevention of STDs. In order to identify a reduced multivariate regression model, stepwise backward selection strategies were performed with the minimum *p*-value for variable selection of 0.2. A *p*-value under 0.05 was regarded as statistically significant. Listwise deletion was used to handle missing data, whereby missing data were simply omitted and the remaining data was analyzed [16].

### 2.4. Ethical Approval

The protocol of the study was approved by the Institutional Review Board of the Vietnam National Hospital of Dermatology and Venereology (code 855/HDDDDBVDLTU).

## 3. Results

In our study, the percentage of female participants was 46.7%. The majority of participants received high school education or higher (88.2%). More than two-thirds of patients lived with spouses/partners (68.4%). In terms of employment status, 39.4% of the participants were white-collar workers, followed by freelancers (28.5%). The mean age was 35.7 years (SD = 12.5).

Table 1 shows the information about the clinical characteristics of the patients. Most participants did not suffer from any STDs. The percentage of patients having genital mycosis was the highest (2%), followed by gonorrhea and syphilis (1.6%). About 9.3% of participants had STDs, and only 1% of participants had STDs related to dermatology. Approximately 10% of participants had dermatological diseases.

Table 2 presents the knowledge of participants regarding STDs. The number of patients who were knowledgeable about syphilis was the highest, followed by herpes warts (57.7%) and HIV/AIDS (57.4%). By contrast, only 17.2% of patients knew that hepatitis C is a STD and about 26.6% of participants considered chlamydia to be an STD. Patients reported that the common symptoms of STDs were purulent genital (53.5%), genital rash (49.6%), and genital ulcers (48.8%). In terms of the curability of STDs, 68% of participants were aware that not all STDs can be cured, and 34.7% of patients knew that several STDs had vaccines. Regarding preventive measures that can be taken against STDs, 80.7% mentioned using condoms.

Participants’ sources of information about STDs are depicted in Table 3. Patients mainly got information about STDs via the Internet (46.1%), friends/relatives (31.9%), and health staff (27.3%). Only 5.1% of participants received information through short message service (SMS)/mobile phone and 8.5% via the local speaker.

Table 4 reveals the factors associated with knowledge about STDs of dermatological patients. Participants who were younger or blue-collar workers were less likely to have adequate knowledge about STDs compared to those who were unemployed or were older. Additionally, participants living with partners had higher scores of STDs knowledge. In terms of information sources, attendants received better information on STDs via the Internet, social networks and health staff compared with those do not use the Internet and/or commute with healthcare providers.

## 4. Discussion

Our study indicated valuable information about knowledge on STDs of dermatological patients in an urban city in Vietnam. The findings showed that a low percentage of participants had adequate knowledge about the common types and symptoms of STDs and about vaccinations against STDs. The majority of patients were aware of the measures of prevention and curability of STDs. Living with partners, having a lower age, and being unemployed (compared to being a blue-collar worker) were positively related to having adequate knowledge about STDs. The Internet, social networks, and health staff were likely to be the most informative sources.

In this study, the percentage of participants who were aware of common types of STDs was relatively low, as opposed to a previous study in which nearly one hundred percent of the participants mentioned HIV/AIDS as being an STD [7]. Compared to the findings of a study conducted among healthcare providers in rural Vietnam, our results also showed a lower rate of knowledge about all common types of STDs [17]. Patients in our study demonstrated a higher level of knowledge about STDs caused by bacteria, such as syphilis and gonorrhea, than STDs caused by viruses (HIV/AIDS and hepatitis). This could be explained by a common belief that viral infections can spread more easily via consuming contaminated food or sharing the same needles rather than sexually [18]. In terms of knowledge about STD symptoms, the majority of patients mentioned purulent genital discharge as being a symptom of STDs, whereas inguinal lymph nodes were often neglected. These findings are consistent with a previous study that revealed that the percentage of participants who were able to correctly provide answers about suspected symptoms was relatively low, and they only identified symptoms presented on the skin [9,19,20].

The majority of patients were aware that using condoms is an effective measure of preventing STDs and HIV. This proportion is higher than the results of previous studies performed in Vietnam [19,21] and a study conducted among young girls in India [22]. However, a study in Ho Chi Minh City showed that approximately 92% of participants were aware that condoms use protects against HIV [23]. There was a disproportion of participants who had adequate knowledge about HIV/AIDS compared to other STDs, and this can be explained by the unbalanced effort and financial resources allocated to combating HIV/AIDS compared to STI control activities in general [24]. This may put patients at a higher risk of becoming infected with other STDs due to inadequate knowledge. The percentage of patients who had misunderstandings about the curability of STDs and the availability of vaccines for STDs was relatively high. A number of STDs are incurable, and the Center for Disease Control and Prevention (CDC) recommends that vaccines are an effective way to prevent hepatitis B and HPV [25].

Our study shows that having a younger age and/or living with a spouse/partner were strongly associated with having better knowledge of STDs. Women who get married typically have more frequent reproductive health examinations [26,27], and are thus more likely to be provided information regarding common symptoms of STDs and STD prevention measures by healthcare providers [19]. Also, unmarried women often hesitate to talk about sexual practices and STDs, probably due to their sensitive nature [26]. A large proportion of STD-infected patients are adolescents [28], since many of them do not perceive themselves to be at risk of becoming infected by an STD [9] and they tend to be more likely to have unprotected sex as well as multiple sexual partners [29]. Therefore, a lot of health promotion campaigns and sex education programs have been established in order to enhance the approachability of STDs information for adolescence. These interventions may increase the knowledge about STDs for young people compared to older people.

Our study also revealed that the Internet, social media, and health care providers were common channels that participants seek for STDs information. This result is similar to a previous study that indicated that the Internet was the most popular source of STDs information [7]. The Internet has upgraded the way that people search for health information, as it can be easily retrieved and passed on [30]. Additionally, the proportion of people using social networks to seek health information also dramatically increased thanks to the ability to receive and respond to information rapidly [31]. A previous study demonstrated that adolescents usually use the Internet to search for their private sexual health problems and collect information on health topics [32]. Nevertheless, as not all the information on the Internet is scrutinized by health professionals, incorrect information may mislead patients and pose the threat of delaying treatments [33].

Several implications can be drawn from our study. In terms of clinical implications, since the lack of understandings about STDs may lead to delays in treatment, health professionals should carefully assess their patients’ knowledge of common types and presentations of STDs, particularly beyond those with skin-related symptoms. Secondly, unmarried women should receive more information about such preventive healthcare service as vaccines in order to protect themselves from STDs and ensure their reproductive health. Several interventions based on the Internet and social media should be undertaken in order to enhance knowledge of STDs, such as peer education, informal conversations within clusters, and/or mass community campaigns. Our findings also provide empirical evidence for decision-makers to assess the effectiveness of current health policies and monitor appropriate adjustments in the future.

Some limitations should be acknowledged. The convenience sampling technique used in this study may limit the ability to interpret the findings from our study. Some of the data that was collected could be incorrect due to recall bias and social desirability bias. In addition, a causal relationship between knowledge of STDs and associated factors cannot be established due to the cross-sectional study design. There was also incomplete data on some variables, which may affect the results of the study.

## 5. Conclusions

This study revealed that there was a low percentage of participants who were adequately aware of the common types, symptoms, and vaccinations available for STDs. Living with spouse/partners and having a younger age were both positively related to having better knowledge about STDs. The use of peer education, informal conversations within clusters, mass community campaigns via the Internet and social networks, and online health care providers should be taken into consideration when designing and implementing interventions.

## Figures and Tables

**Table 1:** Clinical characteristics of respondents. STDs: sexually transmitted diseases.

Characteristic	*n*	%
**Gonorrhea**	10	1.6
**Syphilis**	10	1.6
**Genital mycosis**	12	2.0
**Chlamydia**	6	1.0
**Hepatitis B**	5	0.8
**Human immunodeficiency virus (HIV)**	6	1.0
**Herpes**	3	0.5
**Others**	7	1.1
**Ever had dermatology STDs (*n* = 620)**	10	1.6
**Currently have STDs related to dermatology (*n* = 600)**	6	1.0
**Currently have STDs (*n* = 620)**	58	9.3
**Have more than 1 dermatological disease (*n* = 622)**		
Healthy	564	90.3
One disease	42	6.8
More than 1 disease	16	2.6

**Table 2:** Knowledge of participants towards STDs.

Characteristics	*n*	%
**Types of STDs**		
Syphilis	354	57.8
Herpes warts	353	57.7
Chlamydia	163	26.6
Hepatitis B	172	28.1
Hepatitis C	105	17.2
Human immunodeficiency virus/ acquired immunodeficiency syndrome (HIV/AIDS)	351	57.4
Genital mycosis	258	42.2
Gonorrhea	299	48.9
**Symptoms of STDs**		
Painful or leaky urine	274	44.8
Purulent genital	327	53.5
Genital ulcers	298	48.8
Genital rash	303	49.6
Inguinal lymph nodes	109	17.8
**Preventive measure against STDs**		
Have only one sexual partner	348	56.9
Use condom	494	80.7
Vaccination	143	23.4
Periodic health examination	210	34.3
**Curability of STDs (*n* = 603)**		
Every STD can be cured	48	8
No STD can be cured	28	4.6
Some can be cured, some cannot	410	68
Unknown	117	19.4
**STDs vaccine availability (*n* = 603)**		
Every STD has vaccines	125	20.7
No STDs have vaccines	43	7.1
Some have vaccines, some do not have vaccines	209	34.7
Unknown	226	37.5

**Table 3:** Sources of STDs information.

Characteristics	*N*	%
Friends/relatives	195	31.9
Poster/banner	60	9.8
Internet	282	46.1
Short message service (SMS)/mobile phone	31	5.1
Local speaker	52	8.5
Paper/book	146	23.9
Health staff	167	27.3
Social network	154	25.2

**Table 4:** Factors associated with participants’ knowledge of STDs.

Characteristics	Coef.	95% CI
**Employment** (vs. Unemployed)		
Blue collar worker	−0.34 **	−0.66; −0.03
**Education** (vs. Under high school)		
University/Postgraduate	0.20 *	−0.03; 0.42
**Family income** (vs. Lowest)		
Medium	−0.27 **	−0.51; −0.02
**Marital status** (vs. Single/divorce)		
Living with partners	0.32 **	0.07; 0.58
**Age**	−0.01 **	−0.02; −0.00
**Source of information** (Yes vs. no)		
Internet	0.96 ***	0.74; 1.18
Local speaker	0.20 *	−0.00; 0.41
Paper/book	0.24 *	−0.00; 0.48
Health workers	0.41 ***	0.19; 0.63
Social network	0.24 **	0.01; 0.48
**Currently have STDs** (Yes vs. no)	0.23	−0.12; 0.57

*** *p* < 0.01, ** *p* < 0.05, * *p* < 0.2.
